# Follow-up of pediatric celiac disease: value of antibodies in predicting mucosal healing, a prospective cohort study

**DOI:** 10.1186/1471-230X-14-28

**Published:** 2014-02-13

**Authors:** Edith Vécsei, Stephanie Steinwendner, Hubert Kogler, Albina Innerhofer, Karin Hammer, Oskar A Haas, Gabriele Amann, Andreas Chott, Harald Vogelsang, Regine Schoenlechner, Wolfgang Huf, Andreas Vécsei

**Affiliations:** 1 Department of Pediatrics, Pediatric Gastroenterology, St. Anna Children's Hospital, Medical University Vienna, Kinderspitalgasse 6, 1090 Vienna, Austria; 2 Clinical Department of Pathology, Medical University Vienna, Vienna, Austria; 3 Institute of Pathology and Microbiology, Wilhelminenspital, Vienna, Austria; 4 Department of Internal Medicine III, Division for Gastroenterology and Hepatology, Medical University of Vienna, Vienna, Austria; 5 Department of Food Science and Technology, Institute of Food Technology, University of Natural Resources and Life Sciences, Vienna, Austria; 6 Center for Medical Physics and Biomedical Engineering, Medical University of Vienna, Vienna, Austria

**Keywords:** Pediatrics, Celiac disease, Follow-up, Endomysial antibodies, Sensitivity, Specificity

## Abstract

**Background:**

In diagnosing celiac disease (CD), serological tests are highly valuable. However, their role in following up children with CD after prescription of a gluten-free diet is unclear. This study aimed to compare the performance of antibody tests in predicting small-intestinal mucosal status in diagnosis vs. follow-up of pediatric CD.

**Methods:**

We conducted a prospective cohort study at a tertiary-care center. 148 children underwent esophohagogastroduodenoscopy with biopsies either for symptoms ± positive CD antibodies (group A; n = 95) or following up CD diagnosed ≥ 1 year before study enrollment (group B; n = 53). Using biopsy (Marsh ≥ 2) as the criterion standard, areas under ROC curves (AUCs) and likelihood-ratios were calculated to estimate the performance of antibody tests against tissue transglutaminase (TG2), deamidated gliadin peptide (DGP) and endomysium (EMA).

**Results:**

AUCs were higher when tests were used for CD diagnosis vs. follow-up: 1 vs. 0.86 (*P* = 0.100) for TG2-IgA, 0.85 vs. 0.74 (*P* = 0.421) for TG2-IgG, 0.97 vs. 0.61 (*P* = 0.004) for DPG-IgA, and 0.99 vs. 0.88 (*P* = 0.053) for DPG-IgG, respectively. Empirical power was 85% for the DPG-IgA comparison, and on average 33% (range 13–43) for the non-significant comparisons. Among group B children, 88.7% showed mucosal healing (median 2.2 years after primary diagnosis). Only the negative likelihood-ratio of EMA was low enough (0.097) to effectively rule out persistent mucosal injury. However, out of 12 EMA-positive children with mucosal healing, 9 subsequently turned EMA-negative.

**Conclusions:**

Among the CD antibodies examined, negative EMA most reliably predict mucosal healing. In general, however, antibody tests, especially DPG-IgA, are of limited value in predicting the mucosal status in the early years post-diagnosis but may be sufficient after a longer period of time.

## Background

Celiac disease (CD) is a multi-systemic autoimmune disease triggered by exposure to dietary gluten in genetically predisposed individuals. CD creates small-intestinal mucosal injury of different severity [1]. An effective treatment allowing mucosal healing is the gluten-free diet (GFD). The goals of treatment are not only symptomatic improvement but also avoiding complications, which could arise even in patients having become asymptomatic on a GFD [2,3]. Furthermore, achieving mucosal healing might be crucial because of an increased risk of lymphoproliferative malignancy among patients with persistent villous atrophy [4].

International CD guidelines propose regular follow-up of CD patients [5-7]. Among the follow-up modalities, re-biopsy may be undertaken to prove mucosal healing, which children achieve more often than adults [8]. However, its invasiveness, discomfort and possible complications limit the use of re-biopsy in routine follow-up [5,6]. Therefore, reliable non-invasive surrogate markers of mucosal healing are highly desirable. Whereas antibody tests are of irreplaceable value in diagnosing untreated CD [6], controversy exists over whether these tests can reliably indicate mucosal healing [8-11].

Concerning the correlation between follow-up histology and non-invasive biomarkers, children with CD are an understudied population. Specifically, there is a lack of prospective pediatric studies evaluating current biomarkers used in clinical practice for monitoring purposes.

The purpose of this study was to prospectively compare the performance of up-to-date antibody tests in predicting mucosal status in children with untreated CD vs. in children after prescription of a GFD.

## Methods

### Study design and subjects

**Figure 1:**
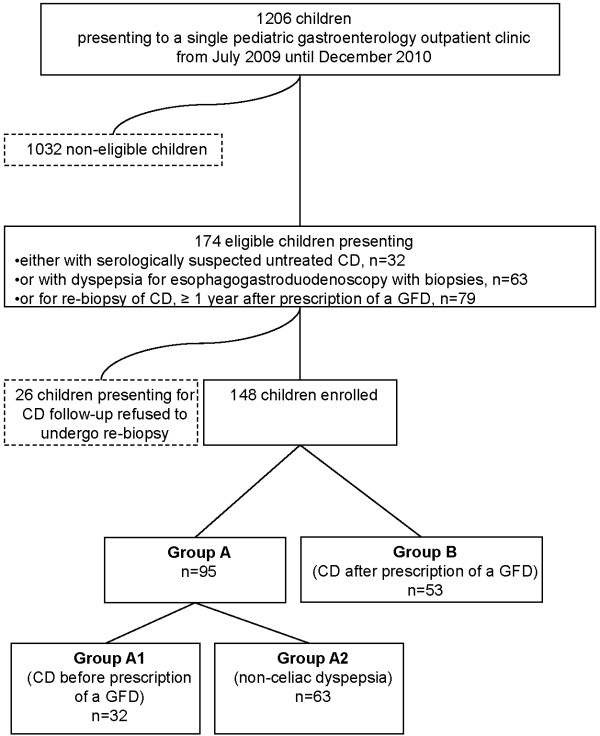
Recruitment flow chart.

Between July 1, 2009, and December 31, 2010, a prospective, cross-sectional cohort study was performed at St. Anna Children’s Hospital. Following written informed parental consent, all consecutively enrolled children (n = 148) underwent esophagogastroduodenoscopy with biopsies (EGD). The participating children were divided into groups according to whether EGD was performed for diagnostic or follow-up purposes (Figure 1).

Group A comprised 95 children on a gluten-containing diet, 32 of them became diagnosed with CD (group A1) and 63 were referred to EGD due to non-celiac dyspepsia (group A2). The predominant complaints in group A1 children were abdominal pain (31.3%), failure to thrive or short stature (18.8%), chronic diarrhea (6.3%), flatulence (6.3%), recurrent headache (6.3%) and constipation (3.1%). A first-degree relative with CD (18.8%), IgA-deficiency (3.1%), autoimmune thyroiditis (3.1%), and iron deficiency anemia (3.1%) were the remaining reasons for CD screening in group A1. Diagnosis of CD was based on positive IgA antibodies against endomysium (EMA) in IgA-competent children or IgG-antibodies against deamidated gliadin peptides (DGP-IgG) in children with IgA-deficiency along with biopsy results consistent with CD (Marsh ≥ 2) and positivity of HLA-DQ2 and/or HLA-DQ8. CD was ruled out by negative biopsy results.

Group B comprised 53 children with CD after prescription of a GFD ≥ 1 year before study enrollment (median 2.2, range 1 to 12.9). CD had been proven by positive EMA or IgA antibodies against tissue transglutaminase (TG2-IgA), biopsy evidence and positivity of HLA-DQ2 and/or HLA-DQ8. Group B children had received regular follow-up according to the recommendations then in force [7]. Within the 18-month study period, a total of 79 children presented for routine CD follow-up. All of these children were invited to participate in the study independent of the presence of symptoms or their adherence to the GFD according to dietary interview. As such they were unselected and only chosen by their willingness to undergo follow-up endoscopy. In this context, 26 of 79 eligible children opted out of the study. The predominant complaints in group B were abdominal pain (15.1%), constipation (1.9%) and aphthous stomatitis (1.9%). Within group B, 79.2% of children were symptom-free.

### Endoscopy, biopsies and histology

All EGDs were performed in anesthesiologist-controlled deep sedation with propofol. Four biopsies were taken from the second part and two from the bulb of the duodenum. Biopsies were staged by two experienced pathologists (GA and AC) who were blinded to subject identity and indication for biopsy. Intestinal histological findings were classified according to a modified Marsh classification [1] using ≥ 30 lymphocytes/100 epithelial cells as cut-off for pathological intraepithelial lymphocytosis [12]. In cases of initial disagreement, a consensus diagnosis was reached using a multihead microscope. Mucosal healing in group A was defined as Marsh < 2.

### CD serology

Blood for serology was taken in the week before EGD. In all children, total IgA levels were determined. IgA-deficiency was defined as serum IgA < 0.07 g/L [13]. All IgA-based tests were evaluated only after exclusion of IgA-deficient children. Four commercial enzyme-linked immunosorbent assays were used for detection of TG2-IgA, TG2-IgG, DPG-IgG, and DPG-IgA (Table 1). Sera were also tested for EMA by indirect immunofluorescence using monkey esophagus (Table 1), at the initial dilution of 1:5 and, when positive, titrated up to the end point. A single experienced technician assessed all slides. CD serology kits from different companies were used because Eurospital TG2-IgA and Orgentec EMA had been our routine test kits since 2005 onwards and on request Werfen Austria, Diagnostic Divisions, was willing to complete the armamentarium of current CD antibodies by providing Inova antibody kits free of charge during the study.

**Table 1:** Antibody tests for celiac disease used in the study

**Assays**	**Manufacturer’s cut-off (U/ml)**	**Optimal ROC cut-off for diagnosing untreated CD (U/ml)**	**Optimal ROC cut-off for monitoring treated CD (U/ml)**
**Endomysial IgA antibodies assay**			
ORG 802/ORG 872 Anti-Endomysium Antibodies kit	1:5	-	1:5
Orgentec Diagnostika GmbH, Mainz, Germany			
**Anti-tissue transglutaminase assays**			
Eu-tTG umana IgA, Eurospital, Trieste, Italy	9	16.4	11.9
Quanta Lite h-tTG IgG ELISA, Inova Diagnostics	20	7.8	10.3
**Antibodies against deamidated gliadin peptide assays**			
Quanta Lite Gliadin IgA II ELISA, Inova Diagnostics, San Diego, USA	20	8.0	8.2
Quanta Lite Gliadin IgG II ELISA, Inova Diagnostics, San Diego, USA	20	11.6	11.9

ROC, receiver operating characteristic; CD, celiac disease; ELISA, enzyme-linked immunosorbent assay.

### Statistics

Data of continuous and categorical variables were reported using median and interquartile range (IQR) on the one hand and counts and percentages on the other hand. For comparisons on categorical data chi-square and Fisher exact test were used while Mann–Whitney U and Kruskal-Wallis tests were used to compare continuous data. Unless otherwise specified, Bonferroni correction was applied for multiple comparisons in post-hoc-tests. Kappa coefficients were used to examine agreement among pathologists in classifying histological findings. Performances of non-invasive tests were evaluated by ROC curve analysis. Areas under ROC curves (AUC), summary measures of overall diagnostic performance, were reported with their 95% confidence intervals (CIs); AUC > 0.9 was considered a high diagnostic test performance [14]. To rank the performance of tests when used for diagnosing CD, AUCs of ROC curves derived from group A were compared [15]. To rank the performance of tests when used for follow-up monitoring of CD, AUCs of ROC curves derived from group B cases were compared. Furthermore, we compared the performance of each single test when used for diagnosis vs. for follow-up monitoring of CD [16]. We also used ROC curve analysis to determine the optimal cut-off point for each test and calculated further performance measures like sensitivity, specificity, positive and negative likelihood-ratios (LR + and LR-) with 95% CIs. Where there was a 2x2 table with an empty cell 0.5 was added to each cell. Tests with either a LR+ > 10 or LR- < 0.1 were considered informative and clinically useful [17]. Statistical calculations were performed with SPSS, version 20.0 (SPSS, Chicago, Ill), and the free software R [18]. For all statistical analyses, Bonferroni corrected two-tailed *P-*values < 0.05 were considered significant.

### Ethics approval

The Institutional Review Board at St. Anna Children’s Hospital approved this study. Since obtaining follow-up biopsies in children with CD deviates from our clinical practice, all group B children were covered by a clinical trial insurance policy valid throughout the 18-month study period.

## Results

### Demographics

Age of the 148 children ranged from 2 to 19 years (median 11.3). Children with newly diagnosed CD (group A1) were younger (median age in years [IQR, min to max]: 7.8 [6.2, 2.0 to 17.6]) than both dyspeptic children (group A2) (median age in years [IQR, min to max]: 12.6 [5.3, 3.3 to 18.0]) and group B children (median age in years [IQR, min to max]: 11.8 [4.8, 4.2 to 19.1]) (*P* < 0.001). In all groups, girls outnumbered boys (girls/boys in group A1: 23/9; in group A2: 48/15; in group B: 33/20) (*P* = 0.26).

### Performance of antibody tests in group A

**Figure 2:**
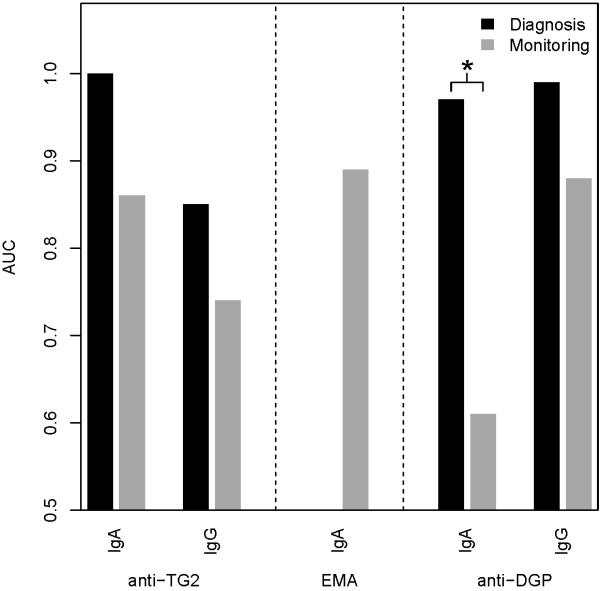
**Areas under ROC curves (AUCs) of antibody tests used in diagnosing untreated coeliac disease vs. monitoring coeliac disease after prescription of a gluten-free diet.** Significant results are marked with asterisks. Anti-TG2 IgA and -IgG, anti-tissue transglutaminase IgA and -IgG; EMA, anti-endomysial antibodies IgA; anti-DGP IgA and -IgG, anti-deamidated-gliadin-peptide IgA and -IgG.

**Figure 3:**
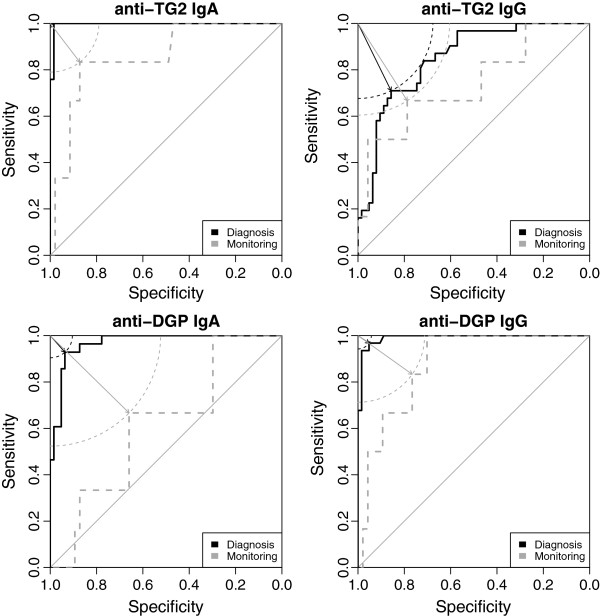
**ROC curves of the antibody tests examined.** In case of anti-TG2 IgA and IgG as well as anti-DGP IgG non-significant differences of AUC between primary diagnosis (solid black line) and follow-up setting (dashed grey line) were found while in anti-DGP IgA (bottom left) significant AUC differences were detected. For illustrative purposes, optimal cut-off points (with minimal distance to the upper left corner) are designated using correspondingly coloured arrows. Circle segments illustrate that the cut-off points chosen are indeed optimal, the diagonal line in each plot represents the general case of a random guess. Perfect discrimination would result in an ROC curve touching the upper left corner (100% Sensitivity, 100% Specificity). AUC, area under the curve; anti-TG2, anti-tissue transglutaminase; anti-DGP, antibodies against deamidated gliadin peptides.

In predicting mucosal status in group A, TG2-IgA, DGP-IgA and DGP-IgG assays showed high diagnostic performance, exhibiting AUCs ≥ 0.96 (Figures 2 and 3, Table 2). TG2-IgG performed less well, exhibiting an AUC of about 0.85.

**Table 2:** Performance of antibody tests in diagnosing untreated celiac disease and monitoring treated celiac disease

**Primary diagnosis of untreated celiac disease (Group A, n = 97)**										
	**Sens**	**95% CI**	**Spec**	**95% CI**	**AUC**	**95% CI**	**LR+**	**95% CI**	**LR-**	**95% CI**
**Anti-tissue transglutaminase**										
Eurospital IgA	1.00	0.85 to 1.00	0.98	0.90 to 1.00	1.00	0.99 to 1.00	41.96	8.62 to 204.13	0.020	0.000 to 0.270
Inova IgG	0.71	0.52 to 0.86	0.86	0.75 to 0.93	0.85	0.77 to 0.93	4.97	2.61 to 9.47	0.340	0.190 to 0.590
**Anti-endomysial antibodies**										
Orgentec IgA	1	0.85 to 1.00	0.85	0.53 to 0.97	-	-	5.51	1.79 to 16.95	0.020	0.000 to 0.320
**Antibodies against deamidated gliadin peptides**										
Inova IgA	0.93	0.76 to 0.99	0.94	0.85 to 0.98	0.97	0.94 to 1.00	14.63	5.63 to 37.96	0.080	0.020 to 0.290
Inova IgG	0.97	0.83 to 1.00	0.95	0.87 to 0.99	0.99	0.98 to 1.00	20.32	6.72 to 61.43	0.034	0.000 to 0.230
**Monitoring treated celiac disease (Group B, n = 53)**										
**Anti-tissue transglutaminase**										
Eurospital IgA	0.83	0.36 to 1.00	0.87	0.74 to 0.95	0.86	0.69 to 1.00	6.53	2.85 to 14.95	0.191	0.032 to 1.147
Inova IgG	0.67	0.22 to 0.96	0.79	0.64 to 0.89	0.74	0.49 to 0.99	3.13	1.42 to 6.90	0.423	0.135 to 1.326
**Anti-endomysial antibodies**										
Orgentec IgA	1.00	0.50 to 1.00	0.74	0.59 to 0.86	0.89	0.80 to 0.98	3.57	2.12 to 5.99	0.097	0.070 to 1.403
**Antibodies against deamidated gliadin peptides**										
Inova IgA	0.67	0.22 to 0.96	0.66	0.51 to 0.79	0.61	0.38 to 0.84	1.96	0.98 to 3.91	0.505	0.160 to 1.596
Inova IgG	0.83	0.36 to 1.00	0.77	0.62 to 0.88	0.88	0.76 to 0.99	3.56	1.90 to 6.68	0.218	0.036 to 1.311

Sens, sensitivity; Spec, specificity; CI, confidence interval; AUC, area under the curve; LR+, positive likelihood-ratio; LR-, negative likelihood-ratio.

Within group A1, all children had positive EMA, positive EMA being an inclusion criterion. Therefore, no performance evaluation for EMA using ROC curve analysis was done in group A.

Within group A2, positive EMA were found in 3 children (specificity 0.94; 95% CI 0.86 to 0.99) who all had Marsh 0 as the result of the small intestinal biopsy. Of these EMA-positive group A2 children, 2 tested positive for TG2-IgA as well. In 2 children, all antibody titres normalized on follow-up under gluten-containing diet within 6 months. One these two children still had Marsh 2 in a follow-up biopsy 28 months later. The third EMA-positive group A2 child was placed on a GFD by her parents for 2.5 years before presenting for follow-up visit. At this visit, she was seronegative. She is currently undergoing a gluten challenge from November 2012 onwards. At her last visit in October 2013 she was still seronegative.

### Performance of antibody tests in group B

Comparing the performance of antibody tests in predicting mucosal status in group A vs. B, all tests performed less well in group B (Figures 2 and 3, Table 2). This performance loss was significant at an uncorrected level of alpha = 0.05 in case of DPG-IgA (*P*_*uncorr.*_ = 0.004). Empirical power (calculated using 1000 bootstrap samples) was 85% for this comparison, and on average 33% (range 13–43) for the non-significant comparisons. Within group B, EMA performed best followed by DGP-IgG and TG2-IgA.

Among the LRs obtained in group B, only the negative LR of EMA was low enough (0.097) to effectively rule out persistent mucosal injury (Table 2).

However, within group B, positive EMA were detected in 18 children, 12 of whom exhibited mucosal healing. Of these children with EMA-positivity despite mucosal healing, all 12 had Marsh 0 as the result of the small intestinal biopsy; 9 (75%) became EMA-negative on further follow-up within the next 20 months. Before study enrollment, all of these 12 children initially had EMA ≥ 1:160 and Marsh ≥ 3A at diagnosis except one girl with EMA 1:80 and Marsh 2. On enrollment, all these children still had positive EMA ranging from 1:5 to 1:80 despite Marsh 0 in the study biopsies after a median of 1.5 years on a GFD. Comparing the duration of the GFD in group B children with mucosal healing, EMA-positive children had been on the GFD for a shorter period of time (median age in years [IQR, min to max]: 1.5 [0.8, 1.0 to 3.0]) than the EMA-negative children (median age in years [IQR, min to max]: 2.3 [5.5, 1.0 to 12.9]) (*P =* 0.008).

Overall, of the group B children exhibiting Marsh 0, 4 had positive TG2-IgA, 15 positive TG2-IgG, 12 positive EMA, 16 positive DPG-IgA, and 10 positive DPG-IgG. All 35 EMA-negative group B children with mucosal healing (34 children with Marsh 0 and one with Marsh 1) also had negative TG2-IgA.

### Histology

In group A1, where all children had histology consistent with CD, severe mucosal injury (Marsh 3B or 3C) was found in 28 children (87.5%) while the remaining 4 children (12.5%) showed Marsh 3a. In group A2, histology showed gastritis in 39 children, esophagogastritis in 8, esophagitis in 1, gastric ulcer in 1 and normal mucosa in 14.

**Figure 4:**
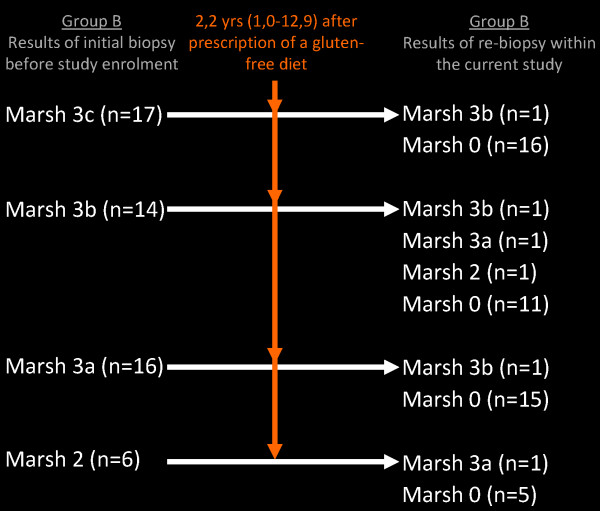
Comparison of histology results of group B at diagnosis (before study enrollment) with the results of re-biopsies taken during the study.

Mucosal healing was found in 47 of 53 (88.7%) group B children (Figure 4, Table 3). Of these children with mucosal healing, 46 showed Marsh 0 (86.8%) and one Marsh 1 (1.8%). Of those children with mucosal injury, one showed Marsh 2 (1.8%), two Marsh 3a (3.8%) and 3 (5.7%) Marsh 3b (Figure 4).

**Table 3:** Comparison of group B children with mucosal healing (Marsh < 2) and mucosal injury (Marsh ≥ 2)

	**Marsh < 2**	**Marsh ≥ 2**	***P***
	**(n = 47)**	**(n = 6)**	
**Serology [n (%)]**			
Negativity of all antibody tests (anti-tissue transglutaminase IgA and -IgG, anti-deamidated-gliadin-peptide IgA and -IgG, anti-endomysial antibodies IgA)	22 (46.8)	0	0.035
Positivity of at least one antibody test	25 (53.1)	6 (100)	
**Chief complaint [n (%)]**			
Asymptomatic	38 (80.9)	4 (66.7)	0.592
Intestinal symptoms	9 (19.1)	1 (16.7)	1.000
Extraintestinal symptoms	0	1 (16.7)	0.113
Weight-for-age *z* score, mean ± standard deviation	−0.66 ± 1.31	−1.04 ± 2.03	0.529
Height-for-age *z* score, mean ± standard deviation	−0.63 ± 1.21	−1.58 ± 1.74	0.094

Good inter-rater reliability among the pathologists was found, kappa coefficients were 0.81 and 0.74 for the biopsies from the bulb and from the descending part of the duodenum, respectively. Before reaching consensus, histological classification differed in 19 children (12.8%); however, in only 2 of these children, classification differences pertained to the presence or absence of mucosal injury (Marsh ≥ 2). The pathologists finally agreed on Marsh ≥ 2 in both children.

No adverse events of endoscopy including biopsies were encountered.

### IgA-deficient children

In total, four children (2.7%), all girls, were IgA-deficient. Three girls belonged to group A1, one to group A2.

## Discussion

In this study, we provide evidence that antibody tests are more reliably predicting mucosal status in children with CD before than in children after prescription of a GFD.

In our patients, according to AUCs (Figure 2), TG2-IgA, DPG-IgG and DPG-IgA antibody tests performed very well in diagnosing CD in group A, similar to the performances reported elsewhere [19]. However, when used for monitoring mucosal status in CD after a median of 2.2 years after primary diagnosis (group B), all tests suffered a performance loss turning out to be significant in case of DPG-IgA. According to LRs, additional parameters quantifying the non-invasive tests' performance, TG2-IgA, DGP-IgG and -IgA antibodies were most informative and clinically useful with respect to diagnosing CD in group A. Conversely, the limited ability to detect mucosal injury in group B was reflected by LRs + in all tests being < 10. Regarding LRs-, only negative EMA had an LR- < 0.1 thus being an informative and clinically useful marker of mucosal healing in CD [17].

Positive EMA, however, were detected in 18 children from group B. Twelve of these EMA-positive children showed mucosal healing, a finding that reflects faster mucosal recovery than EMA-seroconversion. Indeed, EMA-positive children exhibiting mucosal healing had been on the GFD for a significantly shorter period of time than the EMA-negative children. Moreover, 9 of 12 developed EMA-negativity on further follow-up. This delayed seroconversion might partially explain positive EMA in subjects showing mucosal healing [10]. However, adherence to the dietary treatment was not evaluated in this study nor was small intestinal mucosa examined for IgA deposits [20]. Therefore, we cannot rule out that serum EMA-positivity is more sensitive than gross histological damage to detect minor dietary transgressions. Positive EMA without histological evidence of CD were also detected in 3 children from group A2. EMA normalized on a normal gluten-containing diet in 2 of the EMA-positive group B children while the third became seronegative on a self-prescribed GFD and stayed seronegative even after a 11-month gluten challenge. However, since EMA is a very strong predictor of a subsequent CD diagnosis [21], there is need of further follow-up in these children including endoscopy.

All the children participating in the study underwent EGD as criterion standard for the evaluation of the diagnostic reliability of the antibody tests. In the light of the possibility to diagnose CD without biopsies [6], an important finding of our study was that experiences with the diagnostic biopsy did not deter two thirds of the children eligible for group B from undergoing re-biopsy. We found mucosal healing in the majority of the re-biopsied children, 90% of them had Marsh < 2. All group B children with mucosal injury were ≥ 9 years old and belonged to the subgroup that had been on a GFD < 2 years. In children, the long-term mucosal healing rate was reported to be 100% and histological recovery might even occur after more than 2 years after primary diagnosis [22]. In contrast, it has been shown that children diagnosed after the age of 4 tend to follow the GFD less strictly and therefore are expected to have a higher prevalence of mucosal injury [23]. Surprisingly, a very low frequency (1.8%) of isolated increase of intraepithelial lymphocyte count (Marsh 1) was found among group B children. This finding could be related to the somewhat high cut-off for an increased intraepithelial lymphocyte count used in the study (≥ 30 lymphocytes/100 epithelial cells).

This study has several strengths. In the first place, it was conducted in children. Concerning the correlation between follow-up histology and serology, most of the studies investigating this correlation were conducted in adults [2,3,8,9,11,24-27]. However, in one pediatric study on the value of DGP-antibodies in the follow-up of CD, only 13 children had both re-endoscopy and follow-up serology [28]. Another study with children is of limited value in the aspect that only seroconverted children were included [29]. Then, in contrast to the current study, most of the studies reporting on correlations between follow-up histology and serologies are retrospective [2,26,28,29]. Another strength of the current study was the cut-off point being adjusted for the study population in case of all antibody tests according to ROC analysis (Table 1). This adjustment is especially important in children since manufacturers’ cut-offs are usually based on data from adults. A further advantage was the use of AUCs. AUCs as effective single indicators of the agreement between a test and a reference standard facilitate the comparison of the overall performance between different non-invasive diagnostic tests [15,16]. Additionally, in order to increase the reliability of the reference standard, two pathologists, before reaching a consensus diagnosis, independently reviewed all biopsy samples, which had been taken according to guidelines [7,30,31]. Furthermore, we were able to systematically examine all recommended serologic tests. In contrast to recent recommendations for the use of DGP-IgA in monitoring treated CD [5], we found that DGP-IgA suffered a significant performance loss when used for follow-up in children. We therefore consider DGP-IgA less reliable for follow-up purposes compared with EMA, TG2-IgA and DGP-IgG. For the comparisons regarding the performance loss of the serologic tests other than DGP-IgA, mean empirical power was rather low, a finding we consider an important limitation of this study. Therefore, future research is needed to further clarify the correlation of EMA, TG2-IgA and DGP-IgG with follow-up histology or identify other reliable non-invasive follow-up tests in CD.

## Conclusion

In conclusion, this study demonstrates the limited value of serologic testing in the follow-up of pediatric CD with respect to the mucosal status. Only the normalization of EMA indicates mucosal healing with acceptable accuracy. As long as there is a lack of more reliable tools for non-invasive follow-up, EMA should be used as follow-up tool of first choice. However, more reliable non-invasive follow-up tools would be of great clinical and research utility with respect to the individualization of the GFD strictness and upcoming studies evaluating the efficacy of new CD treatment modalities, respectively.

## Abbreviations

CD: Celiac disease; EMA: Endomysial antibodies; TG2: Tissue transglutaminase; DGP: Deamidated gliadin petide; AUC: Area under the curve; GFD: Gluten-free diet; EGD: Esophagogastroduodenoscopy; ROC: Receiver operating characteristic; IQR: Interquartile range; LR-: Negative likelihood-ratio; LR+: Positive likelihood-ratio; max: Maximum; min: Minimum.

## Competing interests

The authors disclose the following: Drs. Edith Vécsei, Stephanie Steinwendner, Hubert Kogler, Albina Innerhofer, Karin Hammer, Oskar A. Haas, Gabriele Amann, Andreas Chott, Harald Vogelsang, Regine Schoenlechner, and Wolfgang Huf have no financial interests to disclose. Dr. Andreas Vécsei has served as a consultant and scientific advisor for Dr. Schär GmbH/Srl, Burgstall, BZ, Italy.

The study was partially supported by Dr. Schär GmbH/Srl, Burgstall, BZ, Italy, and the St. Anna Fund, Vienna, Austria. Antibody kits were supplied free of charge by Inova Diagnostics, Inc., Werfen Group. The study sponsors were not involved in the study design, in the collection, analysis, interpretation of data and in writing of the report.

## Authors’ contributions

EV, SS, HK and AV jointly wrote the first draft of the manuscript. Furthermore, EV developed the study design, the concept and the statistical analysis plan, participated in data collection, interpreted the data and did the statistical analysis and revised the paper; SS developed the study design, the concept and the statistical analysis plan, participated in data collection and entry into the data base, analyzed and interpreted the data; HK cleaned and interpreted the data and participated in statistical analysis and revised the paper and the tables; AI was responsible for the data collection, entry in the database and clinical care of the study patients, critically revised the manuscript for important intellectual content; KH was performing the endoscopies, participated in data collection, was responsible for the clinical care of the study patients, revised the draft paper; OAH was responsible for the serological tests, revised the draft paper; GA and AC reviewed the pathological slides, participated in data collection and revised the draft paper; HV was responsible for the results of the intestinal permeability test and critically revised the manuscript for important intellectual content; RS participated in data collection and revised the paper; WH did the statistical analysis, drafted the figures and critically revised the manuscript for important intellectual content; AV developed the study design, the concept and the statistical analysis plan, obtained the funding, monitored data collection, interpreted the data and did the statistical analysis, drafted and revised the paper and supervised the study. All authors read and approved the final manuscript.

## Pre-publication history

The pre-publication history for this paper can be accessed here:

http://www.biomedcentral.com/1471-230X/14/28/prepub
